# In silico pathway analysis based on chromosomal instability in breast cancer patients

**DOI:** 10.1186/s12920-020-00811-z

**Published:** 2020-11-09

**Authors:** Akeen Kour, Vasudha Sambyal, Kamlesh Guleria, Neeti Rajan Singh, Manjit Singh Uppal, Mridu Manjari, Meena Sudan

**Affiliations:** 1 Human Cytogenetics Laboratory, Department of Human Genetics, Guru Nanak Dev University, Amritsar, Punjab India; 2 Department of Surgery, Sri Guru Ram Das Institute of Medical Sciences and Research, Vallah, Amritsar, Punjab India; 3 Department of Pathology, Sri Guru Ram Das Institute of Medical Sciences and Research, Vallah, Amritsar, Punjab India; 4 Department of Radiotherapy, Sri Guru Ram Das Institute of Medical Sciences and Research, Vallah, Amritsar, Punjab India

**Keywords:** Chromosomal instability, Breast cancer, Pathway analysis

## Abstract

**Background:**

Complex genomic changes that arise in tumors are a consequence of chromosomal instability. In tumor cells genomic aberrations disrupt core signaling pathways involving various genes, thus delineating of signaling pathways can help understand the pathogenesis of cancer. The bioinformatics tools can further help in identifying networks of interactions between the genes to get a greater biological context of all genes affected by chromosomal instability.

**Methods:**

Karyotypic analyses was done in 150 clinically confirmed breast cancer patients and 150 age and gender matched healthy controls after 72 h Peripheral lymphocyte culturing and GTG-banding. Reactome database from Cytoscape software version 3.7.1 was used to perform* in-silico* analysis (functional interaction and gene enrichment).

**Results:**

Frequency of chromosomal aberrations (structural and numerical) was found to be significantly higher in patients as compared to controls. The genes harbored by chromosomal regions showing increased aberration frequency in patients were further analyzed *in-silico*. Pathway analysis on a set of genes that were not linked together revealed that genes *HDAC3, NCOA1, NLRC4, COL1A1, RARA, WWTR1,* and *BRCA1* were enriched in the RNA Polymerase II Transcription pathway which is involved in recruitment, initiation, elongation and dissociation during transcription.

**Conclusion:**

The current study employs the information inferred from chromosomal instability analysis in a non-target tissue for determining the genes and the pathways associated with breast cancer. These results can be further extrapolated by performing either mutation analysis in the genes/pathways deduced or expression analysis which can pinpoint the relevant functional impact of chromosomal instability.

## Background

Complex genomic changes that arise in tumors are a consequence of Chromosomal Instability (CIN), which leads to numerical [(N)-CIN] as well as structural chromosomal instability [(S)-CIN] [1]. The increased levels of aneuploidy and structural complexity in these tumors indicate errors in DNA repair, mitotic segregation and cell cycle checkpoints [2, 3] and may cause (N)-CIN. Structural rearrangements emerge by anomalous DNA repair pathways that cause abnormalities in both homologous and non-homologous end-joining of double-stranded DNA [4, 5]. (S)-CIN may also appear through telomere-mediated events, where decisively short telomeres get identified as DNA breaks capable of recombining (either homologously or nonhomologously) when DNA-repair pathways get compromised and leads to activation of telomerase [6]. The mechanism leading to aneuploidy is distinct from structural changes and aneuploidy arises by disruptions in cell cycle checkpoints and errors in mitotic segregation [2, 7].

CIN is clinically important as it is associated with poor outcome in patients with cancers of lung, breast and colon [8–10] leading to loss or gain of chromosome segments, deletions, translocations, and DNA amplifications [11]. Various studies have reported the correlation between chromosomal aberration and tumor grade and prognosis [8, 12]. Cytogenetic studies in cancer cells have recognized the complexity of genomic rearrangements in cancer cells [13] and have reported recurrent abnormalities in a broad range of tumors [14, 15].

A link between aneuploidy and/or CIN and poor clinical outcome has been identified by several studies [16]. Cancer cells can be targeted based on the whole chromosome instability (W-CIN) phenotype they carry. National cancer Institute (NCI), USA screened compounds having anticancer activity by examining the data-rich drug discovery panel of NCI-60 cancer cell lines and enlisted potential agents with anticancer activity which targeted chromosomally unstable and aneuploid cancer cells [17–19]. NCI also provided a confirmation of the possibility of discovering potential anticancer agents based on the link between their activity and the karyotypic state. An association between aneuploidy and chromosomal instability with distinctive clinical and histopathological features and poor prognosis has also been reported in various cancers [20–22]. Thus, the need to target CIN with new combinatorial strategies has been suggested [23].

Data from large scale genome wide projects have unveiled common core signaling pathways which lead to the development of various cancers [24–28]. Studies to delineate pathways involved in pathogenesis of cancers like colon and glioblastoma multiforme [29], have provided characterization of the genes involved in the pathogenesis of the disease, thus making it significant to focus on pathways which involve various genes [30, 31]. Genomic aberrations disrupt signaling cascades or pathways in tumor cells thereby causing the tumor to proliferate or dedifferentiate uncontrollably [32]. For instance, deletion in any of the components of TGFβ pathway paves way for some of the breast cancers [33–37]. Therapeutic targeting of pathways that are directly involved in initiation of CIN has also gained clinical interest [20, 38]. Pathways-based analysis has gained much importance in the past decade as it is able to, firstly identify the actual genes associated with the phenotype and demarcates them from other false positive hits [39] and secondly marks the biological pathways affected by the genes [40].

The bioinformatics approach can further help in identifying networks of interactions between the genes of interest as well to simultaneously identify biologically informative “linker” genes so as to get a greater biological context of all genes affected by chromosomal instability. This can help to stratify breast cancer patients for choosing optimal treatments and therapies.

Karyotyping aids in efficient single cell screening and identifies important genomic aberrations in normal or diseased samples [41]. A copy number alteration (CNA) is represented by any alteration in banding pattern [42]. This has been indicated by studies which have reported a relation between chromosomal anomalies in peripheral blood lymphocytes (PBLs) and risk prediction in cancers [43–46]. Blood-test screening is considered a non-invasive, cost effective technique [41]. Also, genetic aberrations in a non-target tissue like PBLs may display related events in target tissue [47].

The present study therefore aimed to identify chromosomal anomalies in PBLs of breast cancer patients to: a) identify the recurring aberrant chromosomal lesions and chromosomal loci that are frequently involved in breast cancer; b) determine the genes harboured by these regions, and to delineate the biological pathway which is enriched by them by bioinformatic tools.

## Methods

In the present study 150 patients with confirmed malignant breast cancer were included. The patients were clinically investigated at Sri Guru Ram Das Institute of Medical Sciences and Research, Vallah, Amritsar, Punjab, India. This study was conducted after approval by the institutional ethical committee of Guru Nanak Dev University, Amritsar, Punjab, India. Patients with confirmed malignant breast cancer without any history of any other cancer were included in the study whereas patients having received any kind of therapy (chemotherapy, hormone therapy, radiotherapy or surgery) or blood transfusion, prior to sampling were excluded from the study. After informed consent relevant information including age, gender, occupation, personal history, habitat, habits and disease history were recorded in pre-designed questionnaire. The blood samples of 150 patients and 150 sex and gender matched healthy controls (with no family history of cancer) were collected in a heparinized vial. Peripheral Lymphocyte Culturing was performed by standard 72 h culture method using phytohemagglutinin as mitogen. GTG banding was performed and karyotyping was done following ISCN 2016 [48]. Chromosomal anomalies were assessed in 50–100 metaphases for each subject.

The genes (Table 4) present on the chromosomes involved in anomalies were retrieved from Atlas of Genetics and Cytogenetics in Oncology and Hematology [49] and Genatlas database [50]. On the homepage of Atlas of Genetics and Cytogenetics in Oncology and Hematology, the chromosome number was selected from ‘ENTITIES: by chromosomal band’ and then the genes present on the particular location/band were identified. On the homepage of Genatlas Database, the list of genes present on a particular chromosomal location/band was retrieved by entering the chromosome number and band in “SEARCH in GENATLAS GENES” search field.

**Fig. 1:**
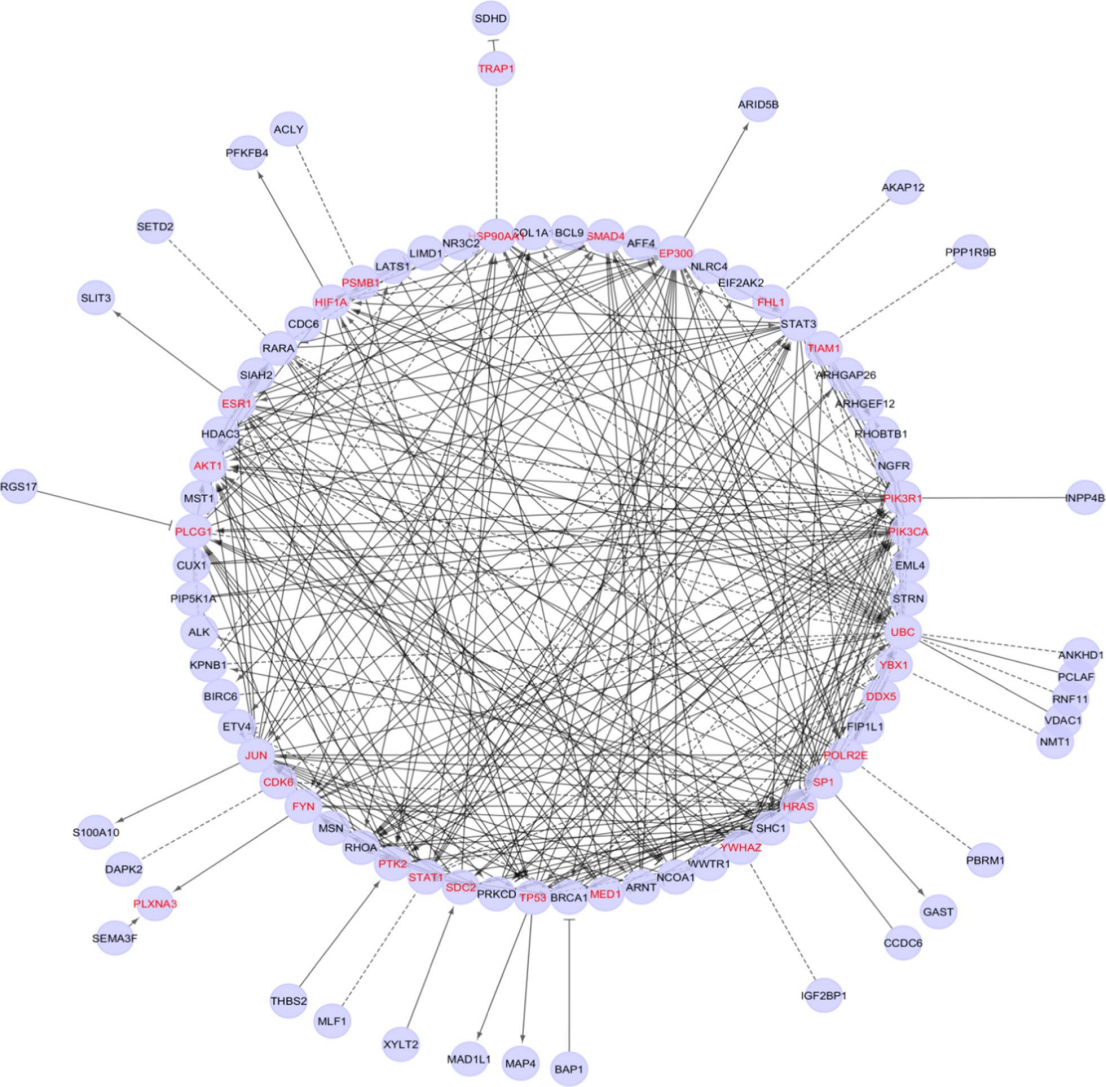
Reactome FI network. *Genes in red font represent the linker genes

Reactome database from Cytoscape software version 3.7.1 was used to perform functional interaction and gene enrichment analysis on the genes (query genes) that were present on the chromosomal regions that were frequently involved in cytogenetic anomalies in the current study. In the Apps menu on Cytoscape software ‘Reactome FI’ was selected. After clicking on this menu, six sub-menus appeared out of which ‘Gene Set/Mutation Analysis’ was selected for performing FI (Functional interaction) analysis on a set of genes. Functional Interaction analysis revealed the involvement of various genes (linker genes) that were linked to the query genes through different networks (Fig. 1).

As majority of the patients (86%) had Infiltrating Ductal Carcinoma (IDCa) of breast, pathway enrichment for invasive ductal breast carcinoma was performed to identify the genes invloved in IDCa. The important features of Reactome FI plug-in were invoked from a pop-up menu by right clicking on the empty space in the network view panel. ‘Load Cancer Gene Index’ option in the pop-up menu opened a list of NCI diseases in the control panel. Here, below ‘Disease Hierarchy’, following options were chosen: Neoplasm—Neoplasm by site—Breast Neoplasm—Malignant Breast Neoplasm—Breast Carcinoma—Breast Adenocarcinoma—Ductal Breast Carcinoma—Invasive Ductal Carcinoma.

Pathway enrichment analysis was further done on a set of genes that are not linked together by checking ‘show genes not linked to others’ in FI Network Construction Parameters. Linkers were not used for pathway enrichment analysis as it leads to bias in results. Right clicking on the empty space in the network view panel led to a pop-menu from which following options were subsequently chosen: Reactome FI – Analyze Network Functions – Pathway Enrichment.

## Results

### Cytogenetic analysis

Cytogenetic analysis was performed on 150 breast cancer patients (147 females and 3 males) and 150 age and gender matched controls. Out of 150 patients, 20 (13.3%), 74 (49.3%), 37 (24.7%) and 14 (9.3%) were diagnosed with stage I, Stage II, stage III and stage IV breast carcinoma, respectively. Exact stage of 5 (3.3%) patients could not be determined. Majority of the patients (89.3%) had IDC of breast. The chromosomal aberrations were counted as in metaphases with: only structural aberrations, with only numerical aberrations and metaphases with both structural and numerical aberrations. A few karyotypes illustrating these aberrations have been provided as Additional file 1: figures (Figure S1-S7). The difference in the frequencies of chromosomal aberrations amongst patients and controls was statistically significant (Table 1). The aberrations were higher in patients as compared to controls: mean (%) aberrant metaphases (22.6 ± 12.3 vs. 12.5 ± 4.6, p < 0.0001), mean (%) metaphases with structural aberrations (11.7 ± 10.8 vs. 4.5 ± 3.1, p** < **0.0001), mean (%) metaphases with numerical aberrations (9.5 ± 6.7 vs. 6.2 ± 3.5; p** < **0.0001). However, mean (%) metaphases with both structural and numerical aberrations were similar in both the groups (2.6 ± 2.0 vs. 2.6 ± 1.1; p = 1.00).

**Table 1:** Cytogenetic profile of breast cancer patients and controls

	Patients	Controls	p-value
No. of subjects	150	150	
Age (Mean ± SD)	50.2 ± 11.5	49.2 ± 14.6	0.51
Mean (%) aberrant metaphases	22.6 ± 12.3	12.5 ± 4.6	** < 0.0001**
Mean (%) metaphases with structural aberrations	11.7 ± 10.8	4.5 ± 3.1	** < 0.0001**
Mean (%) metaphases with numerical aberrations	9.5 ± 6.7	6.2 ± 3.5	** < 0.0001**
Mean (%) metaphases with both structural and numerical aberrations	2.6 ± 2.0	2.6 ± 1.1	1.00
Mean(%) metaphases with acrocentric associations	27.6 ± 14.7	28.9 ± 14.6	0.32

Significant p-value (< 0.05), calculated by t-test, are shown in bold

The stage-wise comparison of cytogenetic profile of breast cancer patients with controls has been shown in Table 2. The chromatid type aberrations observed in patients included premature centromeric division, chromatid break and gap while the chromosome type aberrations included polyploidy, chromosomal gap, pulverizatrion, telomeric associations, chromosomal break, endoreduplication, robertsonian translocations, acentric fragments, ring chromosomes, deletions. Association between the acrocentric chromosome 13, 14, 15, 21 and 22 were scored separately in all metaphases. Acrocentric associations and telomeric bridges were also scored but not counted in the total aberrations. Telomeric associations were commonly seen in acrocentric chromosomes. Apart from acrocentric chromosomes, chromosome 1, 2, 16, 18, 20 and X were also frequently involved in telomeric associations. Breaks and gaps were the most frequent structural chromosomal aberration observed in various regions of different chromosomes. The chromosomes frequently involved in aberrations like loss, gain, deletion, addition and translocations have been shown in Table 3.

**Table 2:** Comparison of cytogenetic profile of Breast cancer patients stage-wise and matched controls

S.No	Patient group and controls	Mean (%) TAM	Mean (%) MSA	Mean (%) MNA	Mean (%) M(SA + NA)#	Mean MAA (%)
1	Stage I cases (n = 20) Controls (n = 20) p-value	20.6 ± 7.6 10.3 ± 3.0 ** < 0.0001**	9.3 ± 5.8 4.7 ± 3.1 **0.0034**	9.2 ± 5.0 4.5 ± 3.2 **0.0011**	3.0 ± 2.3 1.2 ± 1.1 **0.0031**	28.6 ± 13.5 30.2 ± 11.8 0.069
2	Stage II cases (n = 74)* Controls (n = 74) p-value	12.0 ± 9.2 12.9 ± 5.0 0.4609	13.1 ± 9.2 4.7 ± 3.2 ** < 0.0001**	9.4 ± 6.2 6.3 ± 3.4 **0.0002**	2.2 ± 1.5 2.0 ± 1.4 0.4031	28.3 ± 15.0 29.6 ± 15.0 0.5989
3	Stage III cases(n = 37) Control (n = 37) p-value	21.7 ± 12.1 11.9 ± 4.1 ** < 0.0001**	10.6 ± 7.9 4.1 ± 2.8 **0.0015**	9.4 ± 7.5 6.3 ± 3.4 **0.0250**	3.5 ± 2.8 1.5 ± 1.2 **0.0002**	26.0 ± 13.2 18.9 ± 17.9 0.0561
4	Stage IV cases (n = 14) Controls (n = 14) p-value	22.2 ± 9.7 13.8 ± 5.1 **0.0081**	10.9 ± 7.2 5.1 ± 3.5 **0.0117**	10.2 ± 9.2 6.8 ± 3.9 0.2142	2.1 ± 1.0 1.9 ± 1.8 0.7192	31.6 ± 19.3 26.1 ± 18.5 0.4484
5	Indeterminate stage cases (n = 5) Controls (n = 5) p-value	16.7 ± 2.4 13.0 ± 5.5 0.2053	4.7 ± 3.3 3.7 ± 2.2 0.5883	11.5 ± 3.3 7.7 ± 4.8 0.1828	1.0 ± 0.0 1.6 ± 1.5 0.3972	24.6 ± 8.1 26.0 ± 14.0 0.8514

Significant p-value (< 0.05), calculated by t-test, are shown in bold

*TAM* total aberrant metaphases, *MSA* metaphases with structural aberrations, *MNA* metaphases with numerical aberrations, *M(SA + NA):* metaphases with structural and numerical aberrations, *MAA* metaphases with acrocentric associations

^*^One of the subjects with Stage II breast cancer had more than 90% frequency of structural aberration as it was a clonal chromosomal anomaly. Therefore, it was not included in the calculations. Similarly, one of the subjects with Indeterminate stage had a very higher frequency of structural aberrations, thus, it was not included in the calculations

^#^ The zero values were omitted during the calculation of Average and Standard Deviation due to the presence of high number of zero values in Mean(%) M(SA + NA)

**Table 3:** Comparison of frequency of chromosomes involved in various aberrations in the breast cancer patients and controls

Type of aberration	Cases		Controls		p-value
	Chromosomes/ chromosome arms involved	Frequency	Chromosomes/ chromosome arms involved	Frequency	
Loss	5, 8,16, 17, 18, 19, 20, 21, 22, X	78.8 ± 21.20	8, 9, 15, 17, 19, 20, 22, X	57.25 ± 9.23	**0.0169**
Gain	2, 3, 8, 9, X	8.8 ± 4.6	3, 4, 6, 16, 21	9.8 ± 2.28	0.674
Break	1p, 1q, 2p, 2q, 3q, 4p, 4q, 7q, 9q, 17q	12.43 ± 5.29	1q, 2q, 3p, 3q, 4q, 16q	4.4 ± 1.95	**0.0065**
Gap	1p, 1q, 2p, 3p, 3q, 4q, 5q, 6q, 9q, 11q	4.25 ± 2.19	1q, 2q, 5q, 14q, 17q	1.8 ± 0.84	**0.0334**
Deletion	1p, 1q, 2q, 3q, 4q, 5q, Xq	7.2 ± 2.6	1p, 1q, 5p, 6q, Xp	4.2 ± 1.1	**0.0264**
Addition	1q, 9q		9q	NC	NC
Translocations	1, 5, 8, 10, 12, X	4.83 ± 1.47	2, 4, 16	2.66 ± 0.57	**0.0475**
Robertsonian translocation	15, 21	26.5 ± 7.78	13, 21, 22	26.33 ± 3.79	0.9749
Telomeric associations	1, 2, 3, 12,16, 18, 19, 20	9.83 ± 3.37	7,19, X	14.33 ± 4.04	0.1182
	Acrocentric chromosomes:13, 14, 15, 21, 22	101 ± 14.73	Acrocentric chromosomes: 13, 14, 15, 21, 22	44.8 ± 11.19	**0.0008**
Triradials	15, 21, 22	17 ± 5.29	14, 15, 21, 22	9.75 ± 2.36	0.0552

Significant p-value (< 0.05), calculated by t-test, are shown in bold

Chromosomal aberrations present in 2% or more that 2% of metaphases in an individual were considered as clonal anomalies. Both structural and numerical clonal chromosomal anomalies were observed in 28 breast cancer patients (Additional file 1: figure S8, S9). Clonal structural chromosomal anomalies observed in 5 cases were: [(46,XX,add(1)(pter → q21::?::q21 → qter)], [45,XX,del(2)(pter → q11.2::21.2 → qter)],[46,XX,i(21)(q10;q10)], [46,XX,?add(1)(q?21)], [45,XX,t(1;5)(5pter → 5q23::1q25 → qter)]. Noticeably, chromosome 1 was found to be involved in clonal anomalies in three of the cases. Clonal numerical chromosomal anomalies were observed in 23 cases. The most frequent was loss of chromosome X which was observed in 10 cases. Other clonal numerical anomalies included: loss of chromosome 7, 9, 16 and 22 in two cases each; and loss of chromosome 2, 8, 11, 13, 14, 17 and 20 in one case each.

The control subjects had predominantly normal karyotype and the chromosomal aberrations found were lesser in frequency as compared to cases. Moreover, no specific or recurring anomaly was observed in controls. Frequency of non-clonal chromosomal aberrations observed in control were: telomeric association 26.3%; robertsonian translocation 14.3%; premature centromeric division 9.7%; break 9.2%; deletion 8.9%; acentric fragments 8.2%; marker chromosome 5.7%; triradial 3.9%; gap 3.2%; translocation 2.3%; endoreduplication 2.0%; dicentric 2.0%; double minute 0.9%; polyploidy 0.9%; addition 0.7%; ring chromosome 0.5%; fragile site 0.5%; duplication 0.5%; and inversion 0.4%.

To identify the genes harbored by the chromosomal regions showing increased aberration frequency in present study sample, data was retrieved from Atlas of Genetics and Cytogenetics in Oncology and Hematology [49] and Genatlas database [50] (Table 4).

**Table 4:** Genes harboured by the chromosomal regions recurring in anomalies in present study sample

Chromosomal region	Genes^a^
1p32	*RNF11*
1q21	*ARNT, SHC1, PIP5K1A, S100A10, BCL9, MAD1L1, PDE4DIP*
2p21	*EML4*
2p22	*NLRC4, MSN, BIRC6, STRN, EIF2AK2*
2p23	*NCOA1, ALK*
3p21	*LIMD1, MAP4, RHOA, PFKFB4, MST1, SEMA3F, SETD2, PBRM1, BAP1, PBRM1, PRKCD*
3q25	*WWTR1, SIAH2, MLF1, RARRES1*
4q12	*FIP1L1*
4q31	*INPP4B, NR3C2*
5q31	*AFF4, SLIT3, VDAC1, ANKHD1, HDAC3, ARHGAP26*
6q13	*SMAP1*
6q25	*RGS17, AKAP12, LATS1*
6q27	*FGFR10P, THBS2*
7q22	*CUX1*
10q21	*CCDC6, RHOBTB1, ARID5B*
11q23	*SDHD, ARHGEF12*
15q22	*PCLAF, DAPK2*
17q21	*GSDMB, RARA, CDC6, STAT3, GAST, ACLY, BRCA1, ETV4, NMT1, KPNB1, IGF2BP1, NGFR, XYLT2, PPP1R9B, COL1A1*

^a^*Source*: Atlas of Genetics and Cytogenetics in Oncology and Hematology [49] and Genatlas database [50]

#### In-silico analysis

**Fig. 2:**
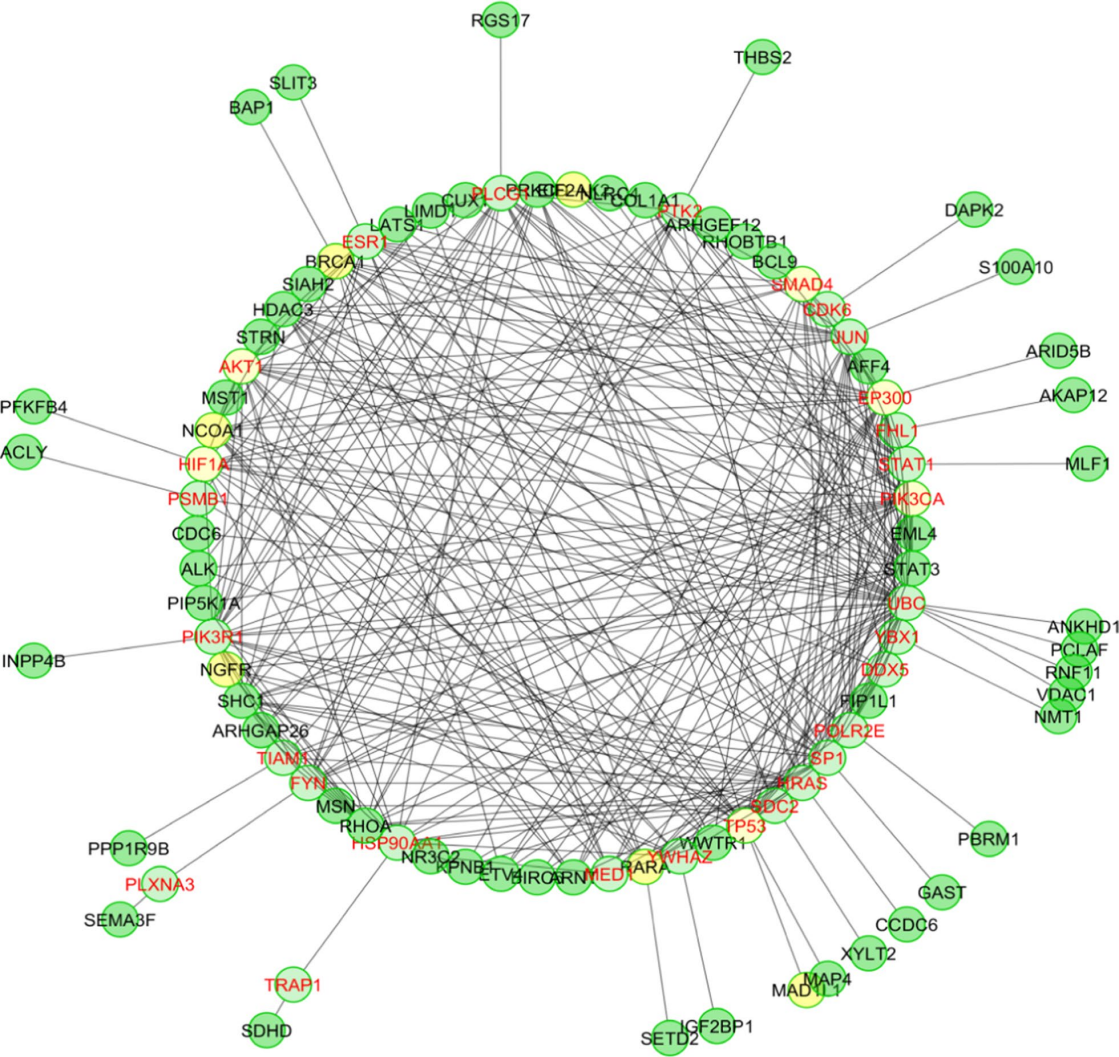
Yellow nodes represent genes enriched in Infiltrating Ductal carcinoma of breast identified by Pathway enrichment

Functional Interaction analysis revealed the involvement of various genes (linker genes) that are linked to the query genes (observed to be harboured by the chromosomal region frequently involved in anomalies in the present study) through different networks (Fig. 1). Pathway enrichment for invasive ductal breast carcinoma was performed to identify the genes invloved in IDC as majority of the patients in the present study sample (89.3%) had IDC of breast (Fig. 2). Linker genes that were involved in IDC were *SMAD4, EP300, PIK3CA, TP53, HIF1A* and *AKT1.*

**Fig. 3:**
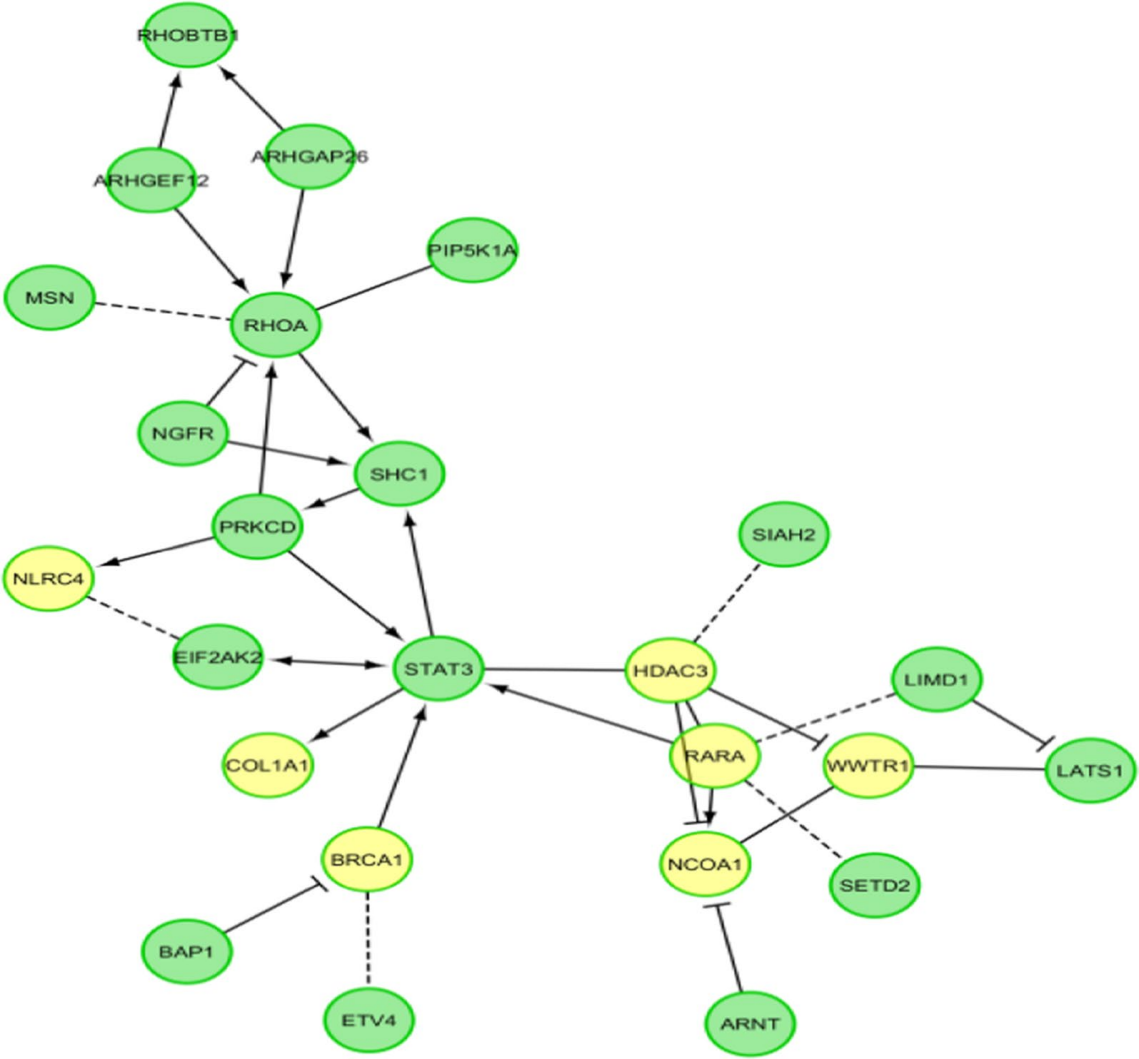
Pathway in FI sub-network. Genes highlighted in yellow color are the ones enriched in RNA polymerase II transcription pathway (p = 0.002, FDR = 0.01)

We analyzed pathways on a set of genes that are not linked together by checking ‘show genes not linked to others’ in FI Network Construction Parameters. Pathway Enrichment analysis revealed that genes *HDAC3, NCOA1, NLRC4, COL1A1, RARA, WWTR1,* and *BRCA1* are enriched in the RNA Polymerase II Transcription pathway (Fig. 3).

## Discussion

Aneuploidy is thought to be a principal outcome of CIN [51]. Chromosomally unstable cancer cells undergo chromosomal missegrgation in excess of every fifth division [52, 53] in contrast to chromosomally stable cells with missegregation occurring in only 1% of cell divisions [52]. Mechanisms that seem to contribute to nCIN are aberrant sister chromatid cohesion [54–57], breach in mitotic checkpoint [58–61], amplification of centrosomes [62] and improper attachment of chromosomes to the mitotic spindle [63, 64]. The whole-chromosome missegregation in mitosis is associated to structural aberrations and DNA damage in the following interphase [65, 66].

Genomes with CIN are characterized by various forms of structural genomic aberrations like amplifications, insertions, reciprocal and non-reciprocal translocations and deletions [5]. In the present study the frequency of various structural (both chromatid type and chromosomal type) and numerical chromosomal aberrations in patients were significantly higher than controls.

Chromosomes that were observed to be frequently involved in aberrations in patients in the present study were 1, 2, 3, 4, 5, 8, 9, 17 and X. Similar aberrations in these chromosomes have been associated with invasive ductal carcinoma of breast and other subtypes [67–69]. Among these, chromosomes 8, 14, 4, 18, X, 3, 10, 20, 9 and 1 have also been observed to contain aberrant regions in breast cancer patients [70].

Large retrospective and prospective studies have given the evidence that the patients having tumors with high aneuploidy have a reduction in recurrence free survival rate that is half as long as those in patients with diploid distribution [71, 72]. Apart from describing the ploidy of DNA content, i.e. diploid or aneuploid, the ploidy-based classification has also been used to understand the degree of genomic instability which reveals the inconsistency of the DNA content in the tumor cell population [73, 74]. In patients with mosaic variegated aneuploidy, premature sister chromatid separation is observed in more than 50% of lymphocytes. In various tissues aneuploidy is seen in more than 25% cells and this enhanced level of aneuploidy leads to higher chances of cancer in these patients [59, 75].

The pathway analysis was performed by Reactome FI to find the linker genes. Pathway enrichment was then performed to further narrow down to the linker genes that were specifically involved in IDC of breast and the genes identified here were *SMAD4*, *EP300*, *PIK3CA*, *TP53*, *HIF1A* and *AKT1*. *SMAD4* has been known to be mainly involved in pancreatic and colorectal cancer [76]. Mutations in *EP300* have been frequently found in skin squamous cell carcinoma and various types of lymphomas [77]. *PIK3CA* has been reported in higher frequency in endometrial, breast and bladder cancers [78]. *TP53* is a tumor suppressor gene and has been found to be mutated in a variety of cancers [79]. As a result of loss of function of various tumor suppressors, the levels of HIF1A increase, indicating that higher HIF1 activity is a common pathway in the pathogenesis of various human cancers [80]. Mutations in regulators of AKT1 signalling pathway have been known to induce oncogenic transformation in human cell. These have been observed mainly in glioma and endometrial cancer but infrequently in cancers like prostate cancer, melanoma, non-small cell lung cancer, breast cancer and hepatocellular carcinoma [81].

Finally, pathway analysis was performed not taking linked genes into account this time. Pathway Enrichment in Analyze Network Functions was performed in Reactome FI application of Cytoscape to find which cellular pathway is enriched by our query genes and the analysis narrowed to 7 genes: *HDAC3, NCOA1, NLRC4, COL1A1, RARA, WWTR1,* and *BRCA1* which were identified to be involved in RNA polymerase II transcription pathway. It was revealed that the genes were significantly enriched in RNA Polymerase II transcription pathway (p = 0.002, FDR = 0.01). RNA Pol II is involved in gene transcription by playing significant role in recruitment, initiation, elongation and dissociation [82, 83]. The role of RNA polymerase II transcription in tumorigenesis has been elucidated in previous studies [84]. It was observed in mouse lymphoma models that tumor cells develop more sensitivity to apoptosis when compared to wild-type cells after treatment with RNA polymerase II transcription inhibitors [85–87]. Enhanced transcription of oncogenes and various transcription factors is associated with transformation in cancer cells [88]. Components of transcriptional apparatus, various oncogenes and ribosomal genes get over expressed in tumor cells in order to maintain proliferation [89–91]. RNPII transcription additionally is required to meet the high need of transcripts like oncogenes and anti-apoptotic factors, which is required to support fast growth and resistance to apoptosis [92].

The role of the genes identified in the current analysis, *HDAC3, NCOA1, NLRC4, COL1A1, RARA, WWTR1,* and *BRCA1*, has already been documented in carcinogenesis. *HDAC3* represses CREB3 mediated transcription and migration of breast cancer cells that are metastatic [93]. *NCOA1* promotes angiogenesis in breast tumors by enhancing the transcription of VEGFa via HIFα and AP-1 [94]. Previous studies from our lab on breast cancer patients from same region have reported association of VEGF polymorphisms + 405C > G, + 936C > T, −2549 Insertion/Deletion, −152G/A, −116G/A, −165C/T and −141A/C with breast cancer risk but no association of VEGF −417C/T, −172C/A and −160C/T and HIF1α polymorphisms (g.C111A, g.C1772T and g.G1790A) with breast cancer risk [95–98].

Majority of the subjects, patients (67.3%) and controls (84%), in the present study were obese with increased central obesity. In the context of obesity, the tumor microenvironment induces an enhanced level of tumor-infiltrating myeloid cells with an activated NLRC4 inflammasome which further activates IL-1b, thus driving progression of disease through adipocyte-mediated VEGFA expression and angiogenesis [99]. Obesity might aid the progression of cancer through the pathways linked with NLRC4 and VEGFA. Thus, prevalence of obesity can have implications for breast cancer risk in the present study sample also.

Cellular expression of *COL1A1* has been reported to possibly promote breast cancer metastasis. This became evident from a study which reported that high levels of COL1A1 were associated with poor survival and a better response to cisplatin-based chemotherapy was observed in ER + breast cancer patients who had increased COL1A1 levels [100]. Breast cancers displaying *RARA* amplifications show sensitivity to retinoic acid [101] and thus these subtypes of breast cancers can be treated with targeted therapies [102]. *WWTR1* also plays a significant role in migration, invasion and carcinogenesis of breast cancer cells [103]. *BRCA1* interacts with a variety of other proteins to carry out multiple functions at cellular level like controlling cell cycle, DNA damage repair, regulation of transcription, replication, recombination and chromatin hierarchical control [104]. In breast cancer patients from same geographical region of north India no association of breast cancer risk with *BRCA1* variants c.190 T > C, 1307delT, g.5331G > A and c.2612C > T was observed [105].

Previous reports have also highlighted the significance of integrative analysis of copy number variations and gene expression profiles in breast cancer [106, 107]. The current study employs the information inferred from chromosomal instability for determining the genes and the pathways associated with breast cancer. The genes/pathways deduced can be further extrapolated by looking for potential mutations that act as key players in breast carcinogenesis. Following up on a lead from the present study, gene expression of the same individuals can be performed. This expression profiling can pinpoint the relevant functional impact of chromosomal instability.

## Conclusion

Breast cancer is a heterogenous disease where mutations in various genes can lead to disease progression. Therefore it becomes important to mark out the cellular pathways involving multiple genes for getting a deeper insight of cancer causation. The present study is a first of its kind where the results of conventional cytogenetics have been exploited to perform gene enrichment analysis. The in silico pathway analysis based on chromosomal instability in PBLs of breast cancer patients hinted towards the RNA polymerase II transcription pathway. Association with breast cancer risk of variants in some of the genes (*p53*, *HIF*, *BRCA1* and *VEGF*) involved in this cellular pathway has been reported from the same population of North India. Further experimental work can help in identifying mutated genes in the pathway and sub-networks to find their relation with breast cancer progression and metastasis.

## Supplementary information

**Additional file 1**. Karyotypes of breast cancer patients.

## Data Availability

The raw datasets generated and/or analyzed during current study are not publicly available in order to protect participant confidentiality. The genes present on the chromosomes involved in anomalies can be accessed from Atlas of Genetics and Cytogenetics in Oncology and Hematology [49] https://atlasgeneticsoncology.org/ and Genatlas Database [50] https://genatlas.medecine.univ-paris5.fr/. On the homepage of Atlas of Genetics and Cytogenetics in Oncology and Hematology, the chromosome number was selected from ‘ENTITIES: by chromosomal band’ and then the genes present on the particular location/band were identified. These genes have the following atlas ID(s) on the Atlas of Genetics and Cytogenetics in Oncology and Hematology- 1p32: RNF11 (44,143); 1q21: ARNT (223), SHC1 (42,287), PIP5K1A (47,397), S100A10 (44,145), BCL9 (466), MAD1L1 (41,226), PDE4DIP (180); 2p21: EML4 (44,353); 2p22: NLRC4 (43,189), MSN (363), BIRC6 (798), STRN (44,243), EIF2AK2 (41,866); 2p23: NCOA1 (44,097), ALK (16); 3p21: LIMD1 (41,158), MAP4 (44,410), RHOA (42,107), PFKFB4 (46,519), MST1 (44,411), SEMA3F (42,254), SETD2 (51,302), PBRM1 (43,697), BAP1 (755), PBRM1 (43,697), PRKCD (42,901); 3q25: WWTR1 (44,545), SIAH2 (46,199), MLF1 (18), RARRES1 (42,050), 4q12: FIP1L1 (40,577); 4q31: INPP4B (43,320), NR3C2 (44,262); 5q31: AFF4 (230), SLIT3 (50,515), VDAC1 (50,902), ANKHD1 (46,476), HDAC3 (40,804), ARHGAP26 (291); 6q13: SMAP1 (42,974); 6q25: RGS17 (47,522), AKAP12 (607), LATS1 (41,127); 6q27: FGFR10P (113), THBS2 (42,549); 7q22: CUX1 (403); 10q21: CCDC6 (280), RHOBTB1 (42,981), ARID5B (51,529); 11q23: SDHD (390), ARHGEF12 (243); 15q22: PCLAF (41,058), DAPK2 (40,263); 17q21: GSDMB (43,972), RARA (46), CDC6 (40,014), STAT3 (444), GAST (44,214), ACLY (50,486), BRCA1 (163), ETV4 (133), NMT1 (43,604), KPNB1 (41,101), IGF2BP1 (40,969), NGFR (41,535), XYLT2 (42,852), PPP1R9B (51,558), COL1A1 (186). On the homepage of Genatlas Database, the list of genes present on a particular chromosomal location/band was retrieved by entering the chromosome number and band in “SEARCH in GENATLAS GENES” search field.

## Abbreviations

CIN: Chromosomal Instability
(N)-CIN: Numerical Chromosomal Instability
(S)-CIN: Structural Chromosomal Instability
NCI: National Cancer Institute
PBLs: Peripheral Blood Lymphocytes
IDC: Infiltrating Ductal Carcinoma
TAM: Total Aberrant Metaphases
MSA: Metaphases with Structural Aberrations
MNA: Metaphases with Numerical Aberrations
M(SA + NA): Metaphases with Structural and Numerical Aberrations
MAA: Metaphases with Acrocentric Associations
