# Supplementary material for: In silico pathway analysis based on chromosomal instability in breast cancer patients
Source: BMC Med Genomics. 2020 Nov 9;13:168. doi: 10.1186/s12920-020-00811-z (PMC7653868; doi:10.1186/s12920-020-00811-z)
Supplement: Supplementary file 1 — Additional file 1. Karyotypes of breast cancer patients. [file 12920_2020_811_MOESM1_ESM.docx]

**Figure S1: Metaphase of a stage I female breast cancer patient showing structural and numerical aberration. Karyotype: 45,XX,add(6)(pter→q27::?),-21**

**Figure S2: Metaphase of a stage II female breast cancer patient showing structural aberration. Karyotype: 46,XX,r(2)(::p25→q37::)**

**Figure S3: Metaphase of a stage II female breast cancer patient showing structural aberration.**

**Karyotype: 46,XX,del(2)(pter→q24:),t(2;13)(13pter→13q34::2q24→2qter)**

**Figure S4: Metaphase of a stage III female breast cancer patient showing structural aberration. Karyotype: 46,XX,inv(1)(p11→p36.3::q11→qter)**

**
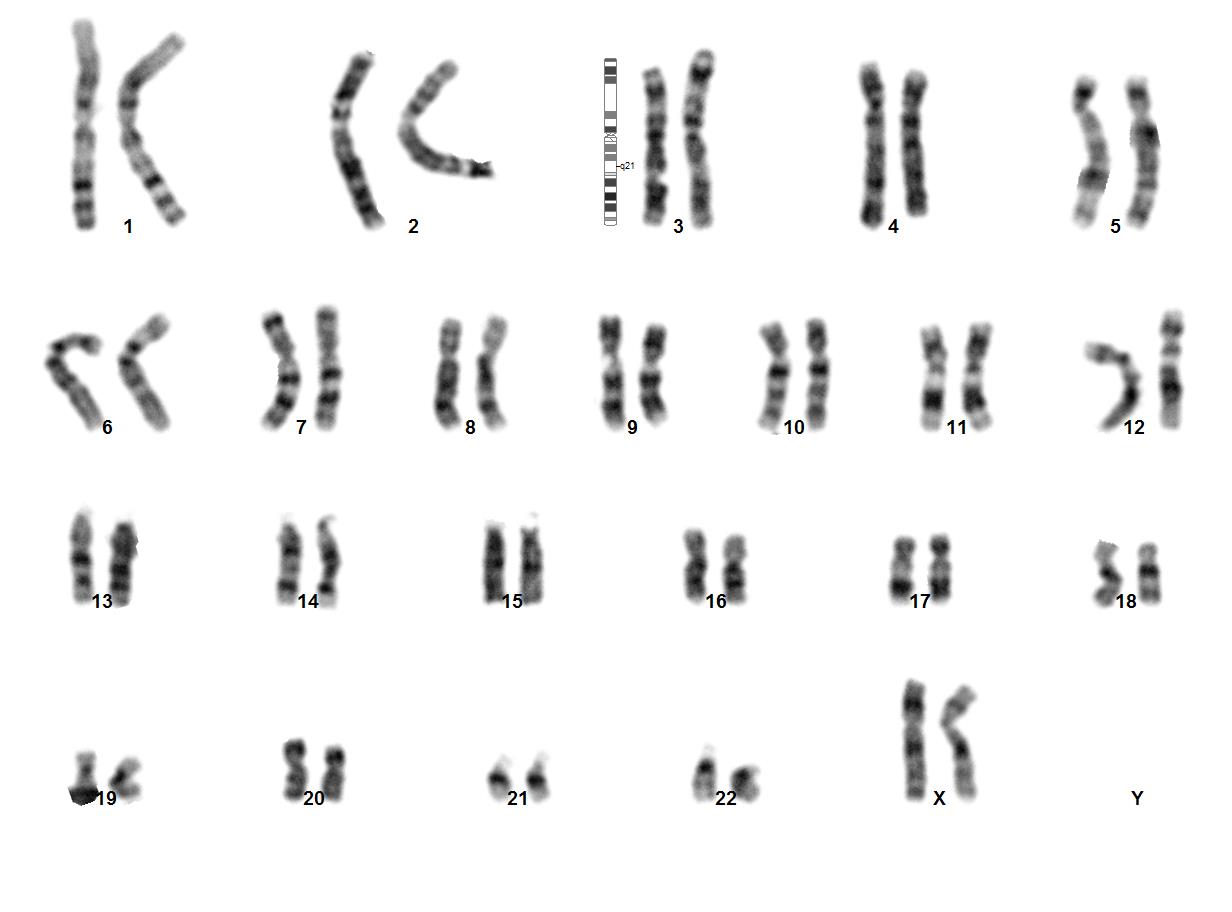
**

**Figure S5: Metaphase of a stage IV female breast cancer patient showing structural aberration. Karyotype: 46,XX,chtb(3)(q21)**

**Figure S6: Metaphase of a male breast cancer patient showing structural aberration.**

**Karyotype: 46,XY,t(X;10)(Xpter→Xq13::10q21→10qter)(10pter→10q11.2::Xq21→Xqter)**

**
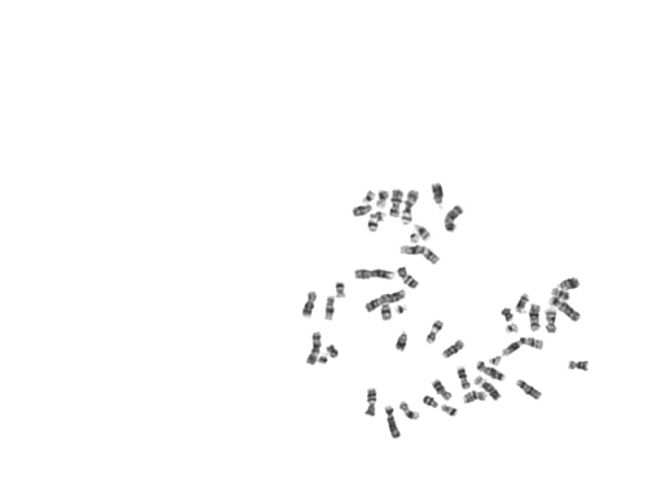
**

**
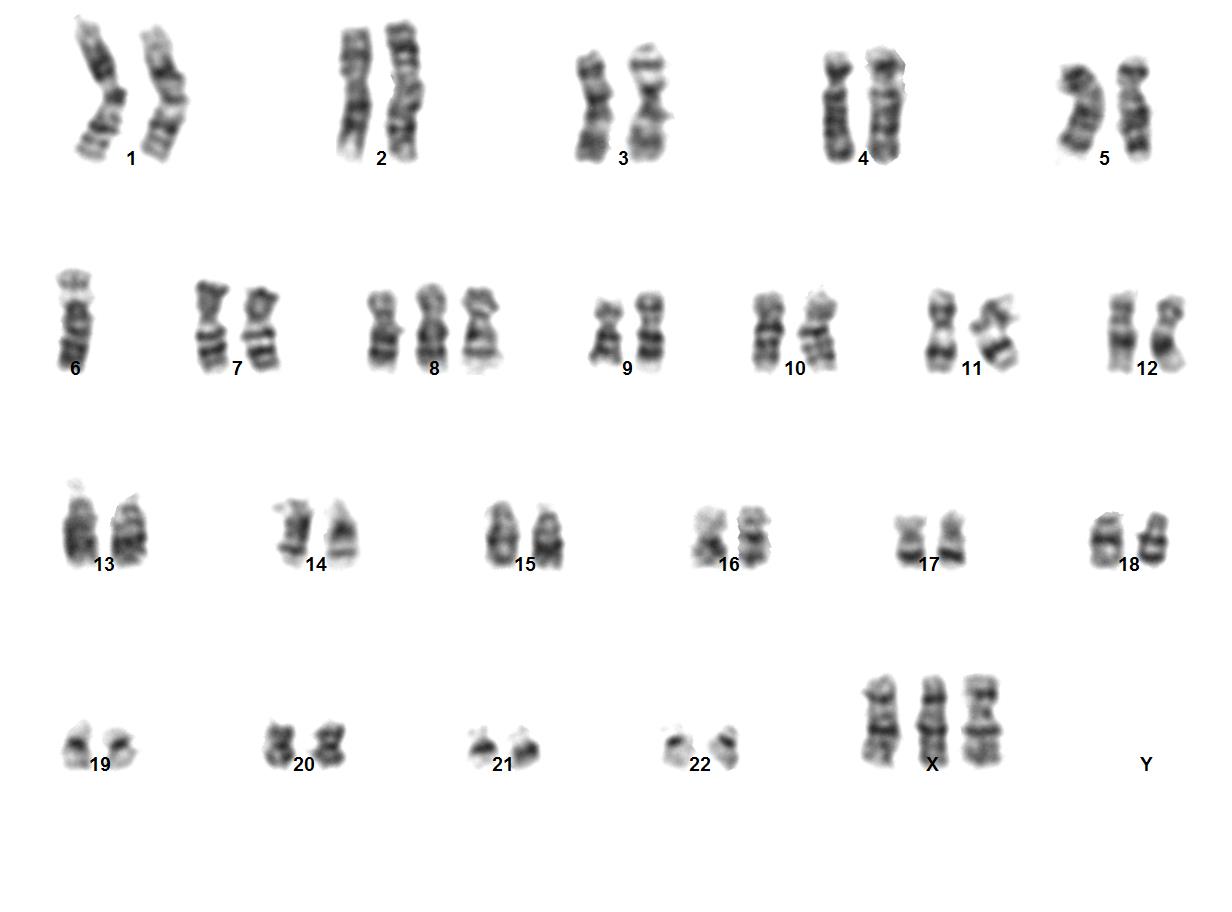
 Figure S7: Metaphase of a stage IV female breast cancer patient showing numerical aberration. Karyotype: 47,XXX,-6,+8**

**Clonal Anomalies:**

**Figure S8: Interstitial deletion in chromosome 2 found in 13% of the scanned metaphases in a stage II female breast cancer patient [46,XX,del(2)(pter→q11.2::21.2→qter)]**

**Figure S9: Addition in chromosome 1q found in 4% of the scanned metaphases in a stage II female breast cancer patient [(46,XX,add(1)(pter→q21::?::q21→qter)]**
